# Clinical effect of anti-resistance exercise combined with nutritional intervention in the treatment of elderly patients with sarcopenia

**DOI:** 10.1097/MD.0000000000039472

**Published:** 2024-09-13

**Authors:** Sheng Chen, Hao Zhang

**Affiliations:** a Department of Orthopedics, Xiantao First People’s Hospital, Xiantao, China; b Department of Orthopedics, Qianjiang Central Hospital, Qianjiang, China.

**Keywords:** grip, nutrition intervention, old age, pace, resistance movement, sarcopenia, skeletal muscle density

## Abstract

This study aims to evaluate the efficacy of a combined intervention involving resistance exercise and nutritional support in improving grip strength, walking speed, and skeletal muscle density among elderly individuals suffering from sarcopenia. Data from a cohort of 500 elderly sarcopenic patients were segregated into observation and control cohorts based on distinct treatment modalities. Baseline evaluations included weight, grip strength, walking speed, and skeletal muscle density. Changes in these parameters and oxidative stress markers were monitored and compared at 1-, 3-, and 6-month intervals. Baseline grip strength for the observation and control groups stood at (20.25 ± 2.34) and (21.06 ± 2.97) kg, respectively. Walking speed was measured at (0.99 ± 0.12) and (0.98 ± 0.20) m/s, respectively. Skeletal muscle density registered (42.98 ± 4.17) and (42.77 ± 5.02) Hu for the observation and control groups, respectively, while muscle mass index was recorded as (6.19 ± 1.46) and (6.20 ± 1.68) kg/m^2^, respectively. Limb skeletal muscle mass for both cohorts was (16.83 ± 3.57) and (16.77 ± 3.89) kg. No significant disparities were discerned in baseline characteristics between the groups. Following 1, 3, and 6 months, the observation group exhibited marked enhancements in grip strength and walking speed (*P* < .05), with substantially superior grip strength compared to the control cohort (*P* < .05). Notably, skeletal muscle density, muscle mass index, and limb skeletal muscle mass exhibited significant augmentation in the observation group (*P* < .05), while no significant alterations were observed in the control cohort. Oxidative stress-related parameters displayed no notable differences between groups pretreatment (*P* > .05). Post-treatment, levels of Hcy, IFN-γ, and MDA markedly decreased in both groups, with considerably lower levels evident in the observation cohort (*P* < .05). Moreover, SOD levels exhibited significant post-treatment increments in both groups, with markedly higher levels observed in the observation group (*P* < .05). An integrated approach of resistance exercise and nutritional support significantly enhances grip strength, walking speed, and skeletal muscle density in elderly patients with sarcopenia, contributing to better prognoses and improved quality of life.

## 1. Introduction

As of now, China’s population aged 60 and over has reached approximately 200 million, classifying it as an aging society. This demographic is projected to grow, potentially surpassing 300 million by 2025. Concurrently, the rise in the elderly population has intensified challenges related to age-associated diseases and health issues, significantly impacting societal, familial, and individual resources. The growing needs for life care, rehabilitation, medical services, and cultural engagement among the elderly are becoming more pronounced.^[1,2]^

Sarcopenia, a critical factor contributing to disability and frailty among the elderly, is characterized by the progressive loss of skeletal muscle mass, strength, and function. Factors such as muscle cell apoptosis, hormonal changes, chronic inflammation, cancer, organ failure, malnutrition, and motor neuron degeneration escalate the risk of developing sarcopenia. This condition is often seen as a precursor to general weakness, compounded by various chronic illnesses, emotional and cognitive impairments, and immunological decline, leading to increased vulnerability and disability in the elderly.^[3,4]^ Evidence indicates that frail elderly individuals, compared to their non-frail counterparts, face higher disability rates, increased mortality, and greater risks of falls, hospital-acquired infections, extended hospital stays, and mortality.^[5,6]^ Given these findings, this study explores the effectiveness of resistance exercise combined with nutritional interventions in improving grip strength, walking speed, and skeletal muscle density in elderly patients with sarcopenia.

## 2. Object and method

### 2.1. Research object

This study was approved by the Ethics Committee of Xiantao First People’s Hospital. In this study, a total of 500 elderly patients with sarcopenia were included, and patients were divided into observation group and control group according to the treatment. Inclusion criteria: patients with sarcopenia aged 65 years or older. Diagnostic criteria for sarcopenia: Decreased muscle mass. Decreased muscle strength. Decreased muscle function. A diagnosis is made if article 1 is satisfied and both 2 and/or 3 are present. Exclusion criteria: disabled and dementia elderly; subjects who were unable to cooperate with the study for various reasons.

### 2.2. Research methods

All participants received standard rehabilitation interventions, which included monitoring of condition, guidance on medication, dietary advice, and routine rehabilitation exercises. Patients were instructed to follow a balanced diet emphasizing foods that are easy to digest and rich in essential nutrients such as proteins and amino acids. The control group was treated with a conventional regimen of aspirin, nitrates, beta-blockers, statins, ACE inhibitors, and other pharmacological therapies. In addition to these treatments, the observation group participated in a tailored program that combined resistance exercise with nutritional enhancements, including resistance training and vitamin D supplementation appropriate for their condition. The specifics of the interventions are detailed below:

In line with the “ACSM Exercise Testing and Prescription Guide,” a tailored resistance training program has been designed, focusing on bodyweight and progressive resistance exercises using resistance bands. The regimen includes exercises like horizontal chest presses, triceps extensions, resistance band pulls, standing leg lifts, seated leg extensions, calf raises, seated kicking, gas pedal-style exercises, and reverse lunges, targeting essential muscle groups such as the quadriceps, hamstrings, glutes, gastrocnemius, infraspinatus, teres minor, forearm, and triceps muscles. Initially, the program involves 1 set of 9 exercises with 10 repetitions each. As the patient progresses, the intensity increases to a moderate level with 2 sets of 9 exercises, maintaining a 30-second rest between sets, conducted twice weekly with a minimum 48-hour rest interval. Each session lasts about 50 minutes, including warm-up and cool-down periods. If symptoms like dizziness or significant shortness of breath occur, the session is halted to ensure safety. This structured regimen aims to enhance muscular strength and functionality, significantly benefiting elderly patients with sarcopenia.

A comprehensive nutritional intervention strategy is employed, adhering to the Chinese Dietary Guidelines (2016 edition). Daily energy and nutritional needs are assessed and tailored to the individual patient, with specific food types chosen to meet these requirements. Nutritional supplementation includes daily doses of whey protein, fish oil, and vitamin D. Whey protein is administered 3 times daily, 10 grams with each meal. Additionally, fish oil and vitamin D are provided in the form of soft capsules, taken twice daily – 2 capsules 30 minutes after breakfast and dinner.

Baseline measurements of weight, grip strength, walking speed, and skeletal muscle density were recorded for both groups, and these parameters were subsequently observed and compared after 1, 3, and 6 months to assess changes. Muscle strength was evaluated using a grip strength test, with grip strength thresholds set at <26 kg for males and <18 kg for females to diagnose decreased muscle strength. The InBody S10 bioelectrical impedance body composition detector was utilized to accurately measure these metrics.

Perform a body composition test. The operator recorded the patient’s name, gender and year, age, height, body mass, etc. The patient then stands barefoot on an empty stomach. Stand in the upright position on the test base of the body composition instrument, with the balls and feet of the front feet on both sides. The heel is respectively in contact with the specified electrode. The patient’s hand holds the electrode and it falls naturally. Lower and separate from the body, completely relax and look ahead, remain quiet until the instrument. Instrument measurement completed. The body index was recorded in the test results, mass index, BMI, skeletal muscle mass (skeletal muscle mass index) and skeletal muscle content of the limbs.

### 2.3. Comparison of relevant indexes of oxidative stress response

Before and after treatment, 5 mL fasting venous blood was extracted from all patients in the morning and centrifuged at the rate of 3000 r/min for 10 min. The upper serum was taken to determine the contents of serum homocysteine (Hcy), interferon-γ (IFN-γ), superoxide dismutase (SOD), and malondialdehyde (MDA). The contents of IFN-γ, SOD. and MDA were determined by enzyme-related immunosorbent assay (ELISA), and the kits and supporting reagents produced by Shanghai Jingkang Biotechnology Co., LTD were used. The Hcy was measured by TC9086 automatic blood biochemical analyzer manufactured by Jiangxi Teckang Technology Co., LTD.

### 2.4. Statistical processing

All the data were statistically processed by SPSS software package and expressed as mean ± standard deviation (*x̅* ± s). Analysis of variance was used for inter-group and The *t* test was used for intra-group comparison, and *P* < .05 showed significant difference. Our continuous variable dataset was assessed for normal distribution using the Shapiro–Wilk test before conducting the main statistical analyses. The *P* values for all major variables were above .05, indicating that the data do not significantly deviate from a normal distribution.

## 3. Results

### 3.1. Distribution characteristics of basic information of subjects

In the study, the gender, age, and body mass index (BMI) of participants in both groups were carefully matched to ensure comparability, and the statistical analysis confirmed that there were no significant differences between the groups in these baseline characteristics. Details of these comparisons are provided in Table 1.

**Table 1:** Distribution characteristics of patients’ basic information.

	Observation group (n = 250)	Control group (n = 250)	*P*
Gender			
Male	98 (39.20)	101 (40.40)	
Female	152 (60.80)	149 (59.60)	.784
Age (years)	79.86 ± 7.54	80.11 ± 7.79	.532
Height (cm)	163.34 ± 5.78	163.01 ± 6.02	.110
Body weight (kg)	61.43 ± 3.97	60.87 ± 3.85	.336
BMI (kg/m^2^)	22.45 ± 1.69	22.29 ± 2.01	.784

*t* test was used for comparison between groups, *P* < .05 indicates statistical significance.

### 3.2. Distribution of baseline disease information

Baseline information showed that the baseline grip strength levels of patients in the observation group and control group were (20.25 ± 2.34) and (21.06 ± 2.97) kg, respectively. The walking speed was (0.99 ± 0.12) and (0.98 ± 0.20) m/s, respectively. Skeletal muscle density was (42.98 ± 4.17) and (42.77 ± 5.02) HU, and muscle mass index was (6.19 ± 1.46) and (6.20 ± 1.68) kg/m^2^, respectively. In addition, the skeletal muscle mass of the limbs was (16.83 ± 3.57) and (16.77 ± 3.89) kg, respectively. Statistical analysis confirmed that there were no significant differences in these baseline parameters between the 2 groups, ensuring comparability. For more detailed information, refer to Table 2.

**Table 2:** Distribution characteristics of baseline information of patients’ disease conditions.

Variable	Observation group (n = 250)	Control group (n = 250)	*P*
Grip strength (kg)	20.25 ± 2.34	21.06 ± 2.97	.427
Walking speed (m/s)	0.99 ± 0.12	0.98 ± 0.20	.498
Skeletal muscle density (HU)	42.98 ± 4.17	42.77 ± 5.02	.447
Muscle mass index (kg/m^2^)	6.19 ± 1.46	6.20 ± 1.68	.943
Limb skeletal muscle mass (kg)	16.83 ± 3.57	16.77 ± 3.89	.699

*t*-test was used for comparison between groups, *P* < .05 indicates statistical significance.

### 3.3. Follow up the distribution of disease changes

**Figure 1.**
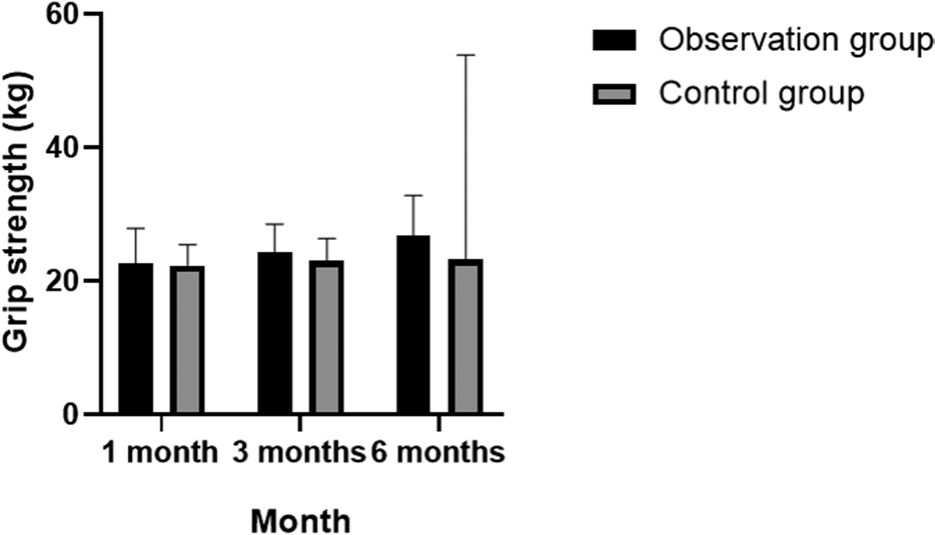
Distribution characteristics of follow-up changes in grip strength of patients.

Following the initial assessments, the relevant health indicators of grip strength and walking speed were measured again at 1, 3, and 6 months. The analysis revealed that patients in the observation group experienced modest improvements in both grip strength and walking speed, with statistically significant differences observed across the 3 time points (*P* < .05). In contrast, the changes in the control group were not statistically significant. After 6 months of follow-up, the grip strength in the observation group was significantly higher compared to that of the control group (*P* < .05). For further details, see Tables 3 and 4; Figure 1.

**Table 3:** Distribution characteristics of follow-up changes in grip strength of patients.

		Observation group (n = 250)	Control group (n = 250)	*P*
Grip strength (kg)	1 mo	22.67 ± 5.22	22.34 ± 3.10	>.05
	3 mo	24.38 ± 4.13	22.98 ± 3.39	>.05
	6 mo	26.70 ± 6.11	23.16 ± 3.07	<.05
	*P*	<.05	>.05	

Analysis of variance was used for inter-group and the *t* test was used for intra-group comparison. *P* < .05 indicates statistical significance.

**Table 4:** Distribution characteristics of patients’ walking speed during follow-up.

		Observation group (n = 250)	Control group (n = 250)	*P*
Walking speed (m/s)	1 mo	1.24 ± 0.43	1.02 ± 0.45	>.05
	3 mo	1.42 ± 0.12	1.12 ± 0.25	>.05
	6 mo	1.53 ± 0.68	1.29 ± 0.61	<.05
	*P*	<.05	>.05	

Analysis of variance was used for inter-group and the *t* test was used for intra-group comparison. *P* < .05 indicates statistical significance.

**Figure 2.**
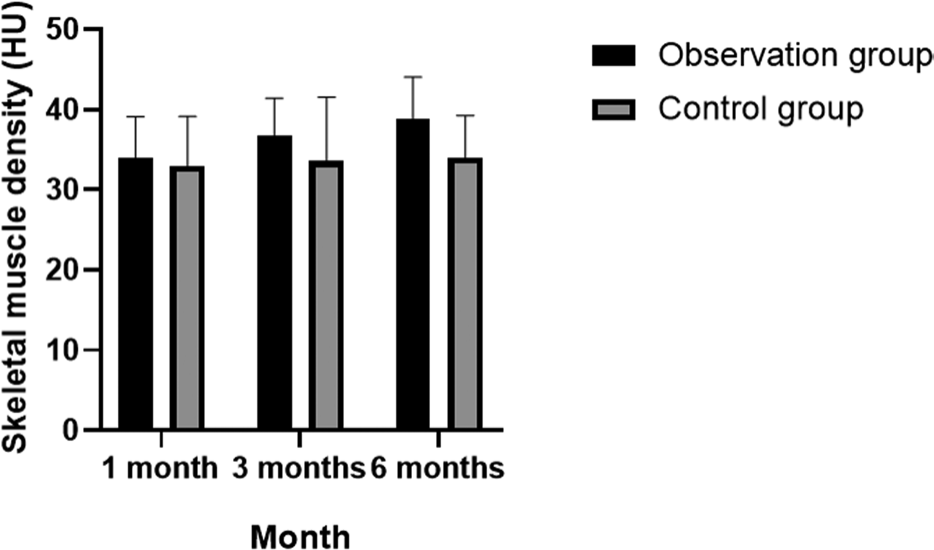
Distribution characteristics of changes in skeletal muscle density during follow-up.

**Figure 3.**
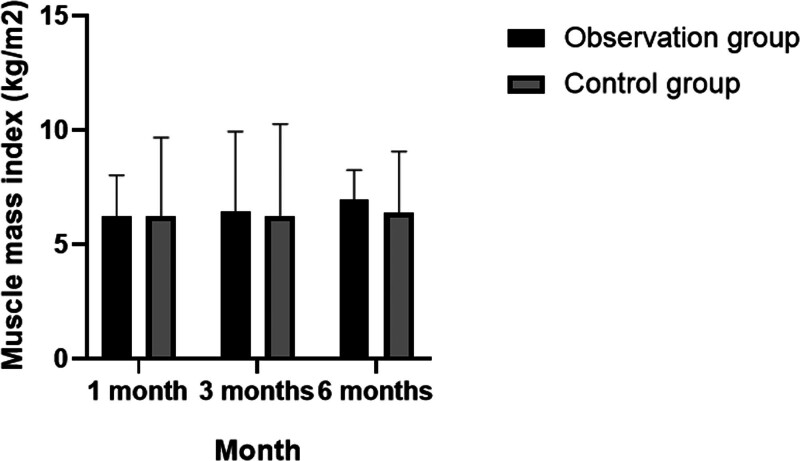
Distribution characteristics of follow-up changes in muscle mass index of patients.

**Figure 4.**
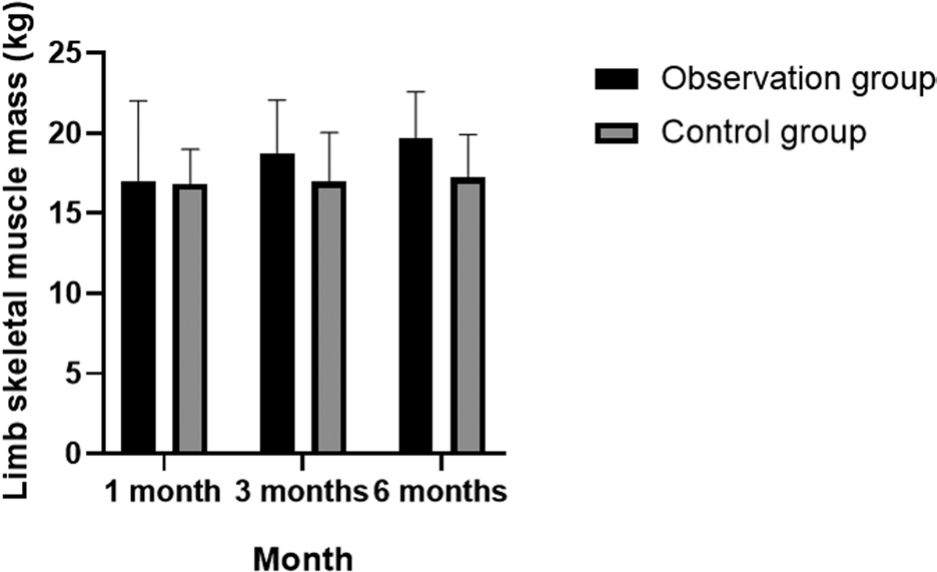
Distribution characteristics of follow-up changes in skeletal muscle mass of patients’ limbs.

The results of this study also indicated that there were significant increases in skeletal muscle density, muscle mass index, and limb skeletal muscle mass levels in the observation group, with statistically significant differences observed across the 3 time points (*P* < .05). In contrast, these metrics showed no statistically significant changes in the control group. Notably, the grip strength of the observation group was significantly higher than that of the control group at both the 3- and 6-month follow-up periods (*P* < .05). For additional details, refer to Tables 5–7; Figures 2–4.

**Table 5:** Distribution characteristics of changes in skeletal muscle density during follow-up.

		Observation group (n = 250)	Control group (n = 250)	*P*
Skeletal muscle density (HU)	1 mo	33.88 ± 5.23	32.99 ± 6.15	>.05
	3 mo	36.76 ± 4.69	33.57 ± 7.98	<.05
	6 mo	38..90 ± 5.15	34.03 ± 5.23	<.05
*P*		<.05	>.05	

Analysis of variance was used for inter-group and the *t* test was used for intra-group comparison. *P* < .05 indicates statistical significance.

**Table 6:** Distribution characteristics of follow-up changes in muscle mass index of patients.

		Observation group (n = 250)	Control group (n = 250)	*P*
Muscle mass index (kg/m^2^)	1 mo	6.24 ± 1.79	6.23 ± 3.45	>.05
	3 mo	6.46 ± 3.48	6.26 ± 4.01	<.05
	6 mo	6.97 ± 1.28	6.41 ± 2.66	<.05
*P*		<.05	>.05	

Analysis of variance was used for inter-group and the *t* test was used for intra-group comparison. *P* < .05 indicates statistical significance.

**Table 7:** Distribution characteristics of follow-up changes in skeletal muscle mass of patients’ limbs.

Limb skeletal muscle mass (kg)	Observation group (n = 250)	Control group (n = 250)	*P*
1 mo	17.04 ± 4.98	16.80 ± 2.19	>0.05
3 mo	18.76 ± 3.29	16.95 ± 3.08	<0.05
6 mo	19.69 ± 2.88	17.21 ± 2.70	*P* < .001
*P*	<.05	>.05	

Analysis of variance was used for inter-group and the *t* test was used for intra-group comparison. *P* < .05 indicates statistical significance.

Before treatment, there were no statistically significant differences between the 2 groups in terms of oxidative stress response indices (*P* > .05). After treatment, significant reductions were observed in the levels of Hcy, IFN-γ, and MDA in both groups compared to pretreatment values, with the observation group showing significantly lower levels than the control group. Additionally, the content of SOD increased significantly in both groups post-treatment, with the observation group demonstrating notably higher levels than the control group. These changes were statistically significant (*P* < .05), indicating a significant difference in the content of oxidative stress-related indices between the 2 groups before and after treatment. For more detailed information, refer to Table 8.

**Table 8:** Comparison of oxidative stress response indexes between the 2 groups before and after treatment (*x̅* ± s).

Group	*n*	Hcy (μmol/L)			IFN-γ (pg/mL)		
		Pretreatment	After treatment	Difference	Pretreatment	After treatment	Difference
Observation group	250	14.64 ± 3.25	11.95 ± 2.46*	2.75 ± 1.12	41.62 ± 3.84	29.23 ± 1.32*	12.64 ± 2.78
Control group	250	14.16 ± 2.94	13.46 ± 2.04*	0.84 ± 1.86	41.74 ± 2.95	34.33 ± 1.81*	8.44 ± 1.35
*t*		0.735	3.170	5.901	0.166	15.272	9.117
*P*		.464	.002	<.001	.868	<.001	<.001

Group	*n*	SOD (U/mL)			MDA (μmol/L)		
		Pretreatment	After treatment	Difference	Pretreatment	After treatment	Difference
Observation group	250	128.16 ± 11.32	149.34 ± 17.65*	21.63 ± 3.32	6.74 ± 0.75	3.58 ± 0.41*	3.39 ± 0.49
Control group	250	127.75 ± 11.19	138.48 ± 14.04*	10.49 ± 2.92	6.67 ± 0.68	4.06 ± 0.58*	2.53 ± 0.55
*t*		0.173	3.230	16.902	0.464	4.533	7.832
*P*		.863	.002	<.001	.644	<.001	<.001

The *t* test was used for intra-group comparison. *P* < .05 indicates statistical significance.

Hcy = Homocysteine, IFN-γ = Interferon-γ, MDA = malondialdehyde, SOD = superoxide dismutase.

## 4. Discussion

The international community has increasingly focused on the concept of healthy aging or successful aging, with the United Nations advocating it as a global objective to address aging issues. Healthy or successful aging is defined as the maintenance of optimal physical, psychological, intellectual, social, and economic functions into old age.^[7,8]^ Research indicates that sarcopenia is a significant contributor to disability and frailty in the elderly.^[9–11]^ This study highlights that sarcopenia can be delayed and its impacts mitigated through early interventions aimed at preserving or restoring elderly functions as much as possible. Such measures can reduce disability and enhance the quality of life for older adults, thus easing the burden on individuals, families, economies, and societies. In regions like Europe, the United States, Japan, and South Korea, the Fried frailty index is utilized for diagnosing sarcopenia. This index includes criteria such as weight loss, reduced grip strength, slow walking speed, low physical activity, or fatigue, with the presence of 3 or more of these criteria confirming a diagnosis. Height adjustment is also considered in the assessment.^[12,13]^

Now our country has entered an aging society, the rapid increase of the elderly, especially the elderly (more than 80 years old), the rapid increase of the frail and disabled old. The contradictions between the elderly’s life care, rehabilitation care, health care, spiritual culture and other needs become increasingly prominent; Foreign studies on frailty and disability are closely related to sarcopenia, but there is no domestic research on sarcopenia at present.^[14–16]^ In this study, weight, grip strength and walking speed were measured to determine the frailty index of the elderly and to further measure the skeletal muscle density of the limbs, so as to obtain early diagnosis and treatment of sarcopenia. In this study, it can be seen that elderly patients with sarcopenia in this study generally have problems such as inadequate grip strength, slow walking speed and low level of musculoskeletal related indicators. Baseline information shows that baseline grip strength levels of patients in the observation group and control group were (20.25 ± 2.34) and (21.06 ± 2.97) kg, respectively. The walking speed was (0.99 ± 0.12) and (0.98 ± 0.20) m/s, respectively. Skeletal muscle density was (42.98 ± 4.17) and (42.77 ± 5.02) HU, and muscle mass index was (6.19 ± 1.46) and (6.20 ± 1.68) kg/m^2^, respectively. In addition, the skeletal muscle mass of the limbs was (16.83 ± 3.57) and (16.77 ± 3.89) kg, respectively. There was no statistically significant difference in baseline information level between the 2 groups, which was fully comparable. This study mainly investigated the positive effects of anti-resistance exercise combined with nutritional intervention, giving patients appropriate anti-resistance exercise, vitamin D and other intervention programs on the elderly with sarcopenia, and indeed proved that such intervention programs can delay the occurrence of asthenia in the elderly, reduce disability, and not only improve the quality of life of the elderly. It also lightens the heavy burden on families and society. In the follow-up of this study, the 1-month follow-up time is still short, and no obvious performance of intervention effect has been seen. However, the significant improvement of various indicators of the subjects has gradually become clear since 3 months, which fully confirms the positive effect of anti-resistance exercise combined with nutrition intervention on elderly patients with sarcopenia.

In this study, relevant indicators including grip strength, walking speed, skeletal muscle density, and muscle mass index were reassessed at 1, 3, and 6 months, followed by detailed statistical analysis. It was observed that the grip strength and walking speed of patients in the observation group improved moderately over these intervals, with statistically significant differences noted at each time point (*P* < .05). In contrast, these changes were not statistically significant within the control group. After 6 months of follow-up, the grip strength in the observation group was notably higher than in the control group (*P* < .05). Additionally, the study found significant increases in skeletal muscle density, muscle mass index, and limb skeletal muscle mass levels in the observation group, with significant differences across the 3 time points (*P* < .05), whereas no significant changes were observed in the control group. The grip strength results at 3 and 6 months further confirmed the observation group’s superior performance compared to the control group (*P* < .05).

In clinical research, Hcy, IFN-γ, MDA, and SOD serve as critical markers of oxidative stress. SOD, a vital endogenous antioxidant enzyme, plays a crucial role in protecting neural tissues by mitigating the harmful effects of oxygen free radicals. A lower level of SOD indicates a higher concentration of oxygen free radicals in brain tissue, leading to more severe neuronal damage. MDA, a byproduct of lipid peroxidation, directly reflects the damage inflicted by oxygen free radicals on brain tissue. IFN-γ, a pro-inflammatory cytokine, helps regulate immune responses by promoting the differentiation of immune cells. Under normal conditions, IFN-γ is minimally expressed in brain tissue; however, its expression significantly increases under oxidative stress conditions such as ischemia and hypoxia, correlating positively with the extent of functional brain damage. Hcy is another common indicator of oxidative stress and can adversely affect cerebral vessels in multiple ways, enhancing the oxidative stress response in brain tissue, exacerbating cerebral ischemia and hypoxia, and diminishing cognitive functions. In this study, before treatment, there were no statistical differences between the 2 groups in oxidative stress-related indices. However, post-treatment analysis revealed significant reductions in Hcy, IFN-γ, and MDA levels in both groups, with the observation group showing significantly greater reductions than the control group. Conversely, SOD levels increased significantly in both groups post-treatment, with the observation group achieving higher levels. These findings suggest that the combination of Ginkgo biloba extract and donepezil can effectively mitigate the oxidative stress response triggered by ischemia and hypoxia in brain tissue.

Sarcopenia poses a significant risk to the elderly, particularly as an early indicator of frailty. This condition often coexists with various chronic diseases, emotional and cognitive dysfunctions, and a decline in immune function, which can lead to increased vulnerability and disability.^[17]^ Studies have shown that at comparable ages, frail elderly individuals have higher rates of disability and mortality compared to their non-frail counterparts. They also face increased risks of falls, hospital-acquired infections, extended hospital stays, and even mortality.^[18–22]^ Currently, there is a lack of domestic research focusing on sarcopenia. In this study, the frailty index in the elderly was assessed by measuring weight, grip strength, walking speed, and the skeletal muscle density of limbs, facilitating the early diagnosis of sarcopenia. Interventions such as resistance exercise, vitamin D supplementation, nutritional support with leucine, and the use of ACE inhibitors and ARB medications have shown effectiveness in enhancing the quality of life for the elderly. These measures not only improve individual well-being but also alleviate the substantial burdens placed on families and society.

## Author contributions

**Conceptualization:** Hao Zhang, Sheng Chen.

**Data curation:** Hao Zhang, Sheng Chen.

**Formal analysis:** Hao Zhang, Sheng Chen.

**Investigation:** Hao Zhang.

**Methodology:** Hao Zhang, Sheng Chen.

**Writing – original draft:** Hao Zhang, Sheng Chen.

**Writing – review & editing:** Hao Zhang, Sheng Chen.

## References

[1] Wu J Huang F Liu C. Effects of resistance exercise on blood glucose level and pregnancy outcome in patients with gestational diabetes mellitus: a randomized controlled trial. BMJ Open Diabetes Res Care. 2022;10:244–6. 10.1136/bmjdrc-2021-002622 PMC8984031 35383101

[2] Amasene M Cadenas-Sanchez C Echeverria I. Effects of resistance training intervention along with leucine-enriched whey protein supplementation on sarcopenia and frailty in post-hospitalized older adults: preliminary findings of a randomized controlled trial. J Clin Med. 2022;11:97. 10.3390/jcm11010097 PMC8745511 35011838

[3] Zhao H Xie Y Zhao M. Effects of moderate-intensity resistance exercise on blood glucose and pregnancy outcome in patients with gestational diabetes mellitus: a randomized controlled trial. J Diabetes Complications. 2022;36:108186. 35379538 10.1016/j.jdiacomp.2022.108186

[4] Zhu X Liu D Zong M Wang J. Effect of swallowing training combined with nutritional intervention on the nutritional status and quality of life of laryngeal cancer patients with dysphagia after operation and radiotherapy. J Oral Rehabil. 2022;49:729–33. 35352383 10.1111/joor.13328

[5] Daniela C Diogo VL Pedro M. MO600: effects of intradialytic progressive resistance and aerobic exercise training on physical function. Nephrol Dial Transplant. 2022;37(Suppl_3).

[6] Loturco I Pereira LA Bishop C. Effects of a resistance training intervention on the strength-deficit of elite young soccer players. Biol Sport. 2022. 10.5114/biolsport.2022.106157 PMC9331327 35959330

[7] Sugiyama H Kobayashi Y Sumida Y. A nutritional intervention that promotes increased vegetable intake in Japanese with non-alcoholic fatty liver disease: a six-month trial. J Clin Biochem Nutr. 2022;70:46–53. 35068681 10.3164/jcbn.21-40 PMC8764111

[8] Martínez-Rodríguez A Cuestas-Calero BJ Martínez-Olcina M Marcos-Pardo PJ. Benefits of adding an aquatic resistance interval training to a nutritional education on body composition, body image perception and adherence to the mediterranean diet in older women. Nutrients. 2021;13:2712. 34444872 10.3390/nu13082712 PMC8400619

[9] Dulal S Prost A Karki S Saville N Merom D. Characteristics and effects of integrated nutrition and stimulation interventions to improve the nutritional status and development of children under 5 years of age: a systematic review and meta-analysis. BMJ Glob Health. 2021;6:e003872. 10.1136/bmjgh-2020-003872 PMC8319976 34321232

[10] Kort A Lydom LN Sailing LN. Nutritional intervention and health-related quality of life in patients undergoing radical cystectomy - a randomized controlled trial. Urol Nurs. 2021;41.

[11] Miller EG Nowson CA Dunstan DW. Recruitment of older adults with type 2 diabetes into a community-based exercise and nutrition randomised controlled trial. Trials. 2016;17:467. 27669823 10.1186/s13063-016-1589-5 PMC5037626

[12] Morishima T Ochi E. Effect of combined aerobic and resistance exercise on serum Klotho secretion in healthy young men -a pilot study. Curr Res Physiol. 2022;5:246–50. 35756695 10.1016/j.crphys.2022.06.001 PMC9218281

[13] Ma C Zhou W Jia Y Tang Q. Effects of home-based Baduanjin combined with elastic band exercise in patients with chronic heart failure. Eur J Cardiovasc Nurs. 2022;21:587–96. 34999764 10.1093/eurjcn/zvab128

[14] Acute effects of the resistance exercise associated with different blood flow restriction pressures on bone remodeling biomarkers. J Exerc Sci Fit. 2022;20:155–60. 35356103 10.1016/j.jesf.2022.02.005 PMC8928066

[15] Correale L Buzzachera F Liberali G. Effects of combined endurance and resistance training in women with multiple sclerosis: a randomized controlled study. Front Neurol. 2021;12:698460. 34421801 10.3389/fneur.2021.698460 PMC8374042

[16] Jang M Park C Tussing-Humphreys L. The effectiveness of sarcopenia interventions for cancer patients receiving chemotherapy: a systematic review and meta-analysis. Cancer Nurs. 2021. 10.1097/NCC.0000000000000957 PMC8627517 34054070

[17] Marziou A Aubert B Couturier C. Combined beneficial effect of voluntary physical exercise and vitamin D supplementation in diet-induced obese C57BL/6J mice. Med Sci Sports Exerc. 2021;53:1883–94. 33787528 10.1249/MSS.0000000000002664

[18] Cheng R Ye XL Qin XJ. Protein supplementation under resistance training conditions in elderly patients with sarcopenia: a meta-analysis of randomized controlled trials. 2021.

[19] Kim DM Kang S. Effects of regulatory resistance exercise on brain neuroplasticity factors in prediabetic elderly. Exerc Sci. 2021;30:88–95.

[20] Yu B Tong S Wu Y Abdelrahim MEA Cao M. Effects of resistance training on exercise ability in chronic obstructive pulmonary disease subjects: a systematic review and meta-analysis. Int J Clin Pract. 2021;75. 10.1111/ijcp.14373 34003587

[21] Bohnert KL Ditzenberger G Bittel AJ. Resistance exercise training with protein supplementation improves skeletal muscle strength and improves quality of life in late adolescents and young adults with Barth syndrome: a pilot study. JIMD Rep. 2021;62:74–84. 34765401 10.1002/jmd2.12244 PMC8574175

[22] Zhao B Wang H. Effect of abdominal breathing combined with brisk walking on intervention effect of female patients with essential hypertension. Glob J Health Sci. 2021;13:76.

